# Task Modulation of Single-Neuron Activity in the Human Amygdala and Hippocampus

**DOI:** 10.1523/ENEURO.0398-21.2021

**Published:** 2022-01-19

**Authors:** Runnan Cao, Alexander Todorov, Nicholas J. Brandmeir, Shuo Wang

**Affiliations:** 1 Lane Department of Computer Science and Electrical Engineering, West Virginia University, Morgantown, WV 26506; 2 Booth School of Business, University of Chicago, Chicago, IL 60637; 3 Department of Neurosurgery, West Virginia University, Morgantown, WV 26506; 4 Department of Radiology, Washington University in St. Louis, St. Louis, MO 63110

**Keywords:** amygdala and hippocampus, dominance, face, human single-neuron recordings, task modulation, trustworthiness

## Abstract

The human amygdala and hippocampus are critically involved in various processes in face perception. However, it remains unclear how task demands or evaluative contexts modulate processes underlying face perception. In this study, we employed two task instructions when participants viewed the same faces and recorded single-neuron activity from the human amygdala and hippocampus. We comprehensively analyzed task modulation for three key aspects of face processing and we found that neurons in the amygdala and hippocampus (1) encoded high-level social traits such as perceived facial trustworthiness and dominance and this response was modulated by task instructions; (2) encoded low-level facial features and demonstrated region-based feature coding, which was not modulated by task instructions; and (3) encoded fixations on salient face parts such as the eyes and mouth, which was not modulated by task instructions. Together, our results provide a comprehensive survey of task modulation of neural processes underlying face perception at the single-neuron level in the human amygdala and hippocampus.

## Significance Statement

The human amygdala and hippocampus play important roles in face perception, but it remains unclear how task demands or evaluative contexts modulate neural face processing, especially at the single-neuron level in the human brain. In this study, we comprehensively analyzed how task instruction modulates key aspects of face processing, including low-level facial features such as face shape and texture, social trait judgment of faces such as trustworthiness and dominance, as well as neural correlates of eye movement when viewing faces. Our comprehensive survey of task modulation of face processing reveals both flexible and invariant neuronal processes in the human brain.

## Introduction

The human amygdala and hippocampus are critically involved in face perception (Adolphs, 2008; Todorov, 2012; Rutishauser et al., 2015a; Montagrin et al., 2018). They play several important roles in face processing.

First, the human amygdala and hippocampus encode social judgment of faces. Single neurons in the human amygdala and hippocampus not only encode facial emotions (Fried et al., 1997), but also subjective judgments of facial emotions (Wang et al., 2014), as well as ambiguity in facial emotions (Wang et al., 2017). In addition, both lesion (Adolphs et al., 1998) and functional neuroimaging (Todorov et al., 2008) studies have shown that the amygdala plays an important role in encoding perceived facial trustworthiness. Our recent data have suggested that there exists a neuronal population code for a comprehensive social trait space in the human amygdala and hippocampus (Cao et al., 2021a).

Second, single neurons in the human amygdala and hippocampus encode memory of faces (Rutishauser et al., 2015b) and demonstrate category-selective response to faces (Kreiman et al., 2000), a key function of the amygdala and hippocampus that is supported by forming a highly sparse representation of identity at the level of single neurons. Although a classic model for face representation in the amygdala and hippocampus argues for two prominent properties: (1) the representation of identities is invariant to visual features (Quian Quiroga et al., 2005; Quian Quiroga, 2012); and (2) identity neurons encode conceptually related (e.g., Bill Clinton and Hillary Clinton) but not visually related identities (De Falco et al., 2016; Rey et al., 2020), our recent data have demonstrated that neurons in the human amygdala and hippocampus also embody feature-based coding of faces (Cao et al., 2020b), a mechanism that bridges the perception-driven representation of facial features in the higher visual cortex and the memory-driven representation of semantics in the amygdala and hippocampus.

Third, the human amygdala and hippocampus process information in the eyes and mouth and direct eye movements to these salient face parts (Cao et al., 2021b). Neuroimaging studies have shown that amygdala activation predicts gaze direction (Gamer and Büchel, 2009) and monkey studies have shown that amygdala neurons encode not only the eyes but also the gaze direction when viewing a monkey face, as well as eye contact with the viewed monkey (Hoffman et al., 2007; Mosher et al., 2014).

However, crucial questions remain: are these aspects of face processing modulated by explicit task demands or the evaluative context? If so, which and how are these processes dynamically modulated by tasks and contexts? Our own prior neuroimaging studies have not only found context-independent neural responses in the amygdala to facial trustworthiness during approach versus avoidance decisions (Wang et al., 2018a), but also context-dependent neural responses in the amygdala to facial trustworthiness during face evaluations (Cao et al., 2020a; see also Todorov et al., 2011) for both task-dependent and task-invariant neural responses to facial trustworthiness in the amygdala and hippocampus), indicating that different aspects of face processing may be subject to context modulations differently. Furthermore, a recent study has shown that neurons in the human amygdala and hippocampus are flexibly engaged in memory retrieval (Minxha et al., 2020). In the current study, we conducted a comprehensive survey of task modulation on different aspects of face processing at the single-neuron level in humans. We test the hypothesis that task instructions modulate representation of social traits but not low-level facial features. We also explored the extent to which eye movement was modulated by task instructions.

## Materials and Methods

### Participants

Six neurosurgical patients (two male, 26–47 years old; Table 1) undergoing epilepsy monitoring participated in this study. All neural recording sessions had simultaneous eye tracking. All these participants provided written informed consent using procedures approved by the Institutional Review Board of the West Virginia University.

**Table 1:** List of patients

ID	Age	Sex	Race	Epilepsy diagnosis	Trustworthiness task						Dominance task					
					Number of amygdala neurons			Number of hippocampal neurons			Number of amygdala neurons			Number of hippocampal neurons		
					Total	Left	Right	Total	Left	Right	Total	Left	Right	Total	Left	Right
P6	33	F	White	Left posterior neocortical extratemporal/parietal	6	6	0	20	20	0	9	9	0	27	27	0
P7	28	F	White	Right mesial temporal	3	3	0	23	21	2	8	8	0	36	36	0
P10	47	F	White	Right mesial temporal and neocortical temporal	22	0	22	0	0	0	3	0	3	0	0	0
P11	33	F	White	Right mesial temporal and extratemporal	17	0	17	0	0	0	15	0	15	0	0	0
					7	0	7	0	0	0	12	0	12	20	0	20
					5	0	5	12	0	12	3	0	3	11	0	11
P14	26	M	White	Bilateral amygdylar/ hippocampal	22	18	4	7	5	2	23	18	5	7	5	2
					23	20	3	8	6	2	27	22	5	8	6	2
					20	17	3	32	31	1	21	18	3	41	40	1
					11	9	2	6	4	2	12	10	2	5	3	2
P15	37	M	White	Left amygdylar/ hippocampal	11	11	0	3	0	3	22	22	0	13	0	13
					12	12	0	20	0	20	14	14	0	3	0	3
Sum					159	96	63	131	87	44	169	121	48	171	117	54

Each row of neurons represents a separate recording session. Total: all neurons recorded from an area that had a FR greater than 0.15 Hz. Left, Neurons that were recorded from the left side of an area. Right, Neurons that were recorded from the right side of an area.

### Stimuli

We used the FaceGen Modeller program (http://facegen.com; version 3.1) to randomly generate 300 faces (for detailed procedures, see Oosterhof and Todorov, 2008). FaceGen constructs face space models using information extracted from 3D laser scans of real faces. To create the face space model, the shape of a face was represented by the vertex positions of a polygonal model of fixed mesh topology. With the vertex positions, a principal component analysis (PCA) was used to extract the components that accounted for most of the variance in face shape. Each PC thus represented a different holistic nonlocalized set of changes in all vertex positions. The first 50 shape PCs were used to construct faces that had a symmetric shape. Similarly, because skin texture is also important for face perception, 50 texture PCs based on PCA of the RGB values of the faces were also used to represent faces. The resulting 300 faces were randomly generated from the 50 shape and 50 skin texture components with the constraint that all faces were set to be white. It is worth noting that each PC is a feature dimension of the face space.

Notably, we have already acquired trait judgments of these faces from healthy control raters on nine social traits (Oosterhof and Todorov, 2008): attractiveness, competence, trustworthiness, dominance, mean, frightening, extroversion, threatening, and likability. The trait judgements were measured on nine-point scales, ranging from 1 [not at all (trait)] to 9 [extremely (trait)]. Therefore, these faces have benchmark ratings, and we can readily perform correlational analysis with neural responses and psychometric behavioral data.

### Experimental procedure

**Figure 1.**
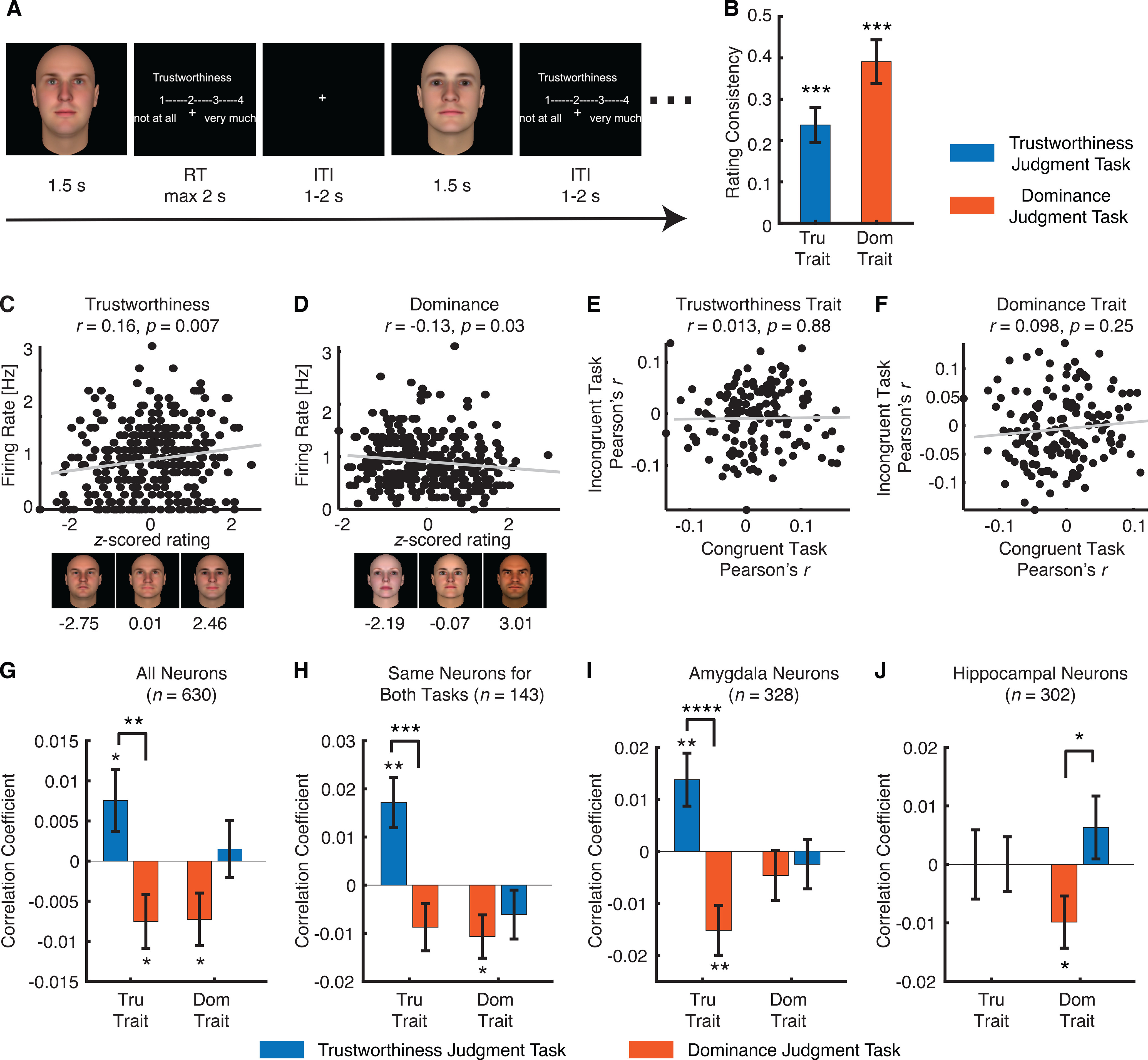
Task modulation of neural encoding of social traits. ***A***, Task. Each face was presented for 1.5 s, followed by participants’ judgment of trustworthiness/dominance within 2 s. The overall intertrial interval was jittered between 1 and 2 s. ***B***, Correlation between the ratings from our neurosurgical patients with consensus ratings from Oosterhof and Todorov (2008). Tru: trustworthiness. Dom: dominance. Error bars denote 1 SEM across sessions. Asterisks indicate a significant difference from 0 using two-tailed paired *t* test; ***p < 0.001. ***C***, ***D***, Example neurons that showed a significant correlation between the normalized FR and the *z*-scored rating for (***C***) the trustworthiness trait and (***D***) the dominance trait. Each dot represents a face image, and the gray line denotes the linear fit. Sample face images with a range of consensus trustworthiness/dominance ratings are illustrated below the correlation plots, and the corresponding consensus ratings (*z*-scored) are shown under each sample face image. ***E***, ***F***, Scatterplot of correlation coefficient between congruent versus incongruent conditions. Each circle represents a neuron, and the gray line denotes the linear fit. ***E***, Trustworthiness trait. ***F***, Dominance trait. ***G–J***, Correlation between neural response and social judgment ratings. Bars show the mean correlation coefficient under each task instruction. Error bars denote ±SEM across neurons. Asterisks indicate a significant difference from 0 (two-tailed paired *t* test) or between conditions (two-tailed two-sample *t* test); *p < 0.05, **p < 0.01, ****p < 0.0001. For each trait (trustworthiness or dominance), the left bars show the congruent conditions (i.e., the evaluated traits were the same as the task instruction) and the right bars show the incongruent conditions (i.e., the evaluated traits were different to the task instruction). ***G***, All neurons (*n *=* *630). ***H***, The same neurons that were recorded in both trustworthiness and dominance judgment tasks (*n *=* *143). ***I***, Amygdala neurons only (*n *=* *328). ***J***, Hippocampal neurons only (*n *=* *302). Extended Data Figure 1-1 shows assessment of spike sorting and recording quality. Extended Data Figure 1-2 shows control analyses for choices.

For FaceGen stimuli, participants performed two face judgment tasks: trustworthiness judgment task and dominance judgment task (Fig. 1*A*). In each task, there was a judgment instruction, i.e., participants judged how trustworthy or how dominant a face was. We used a 1–4 scale: 1: not trustworthy/dominant at all; 2: somewhat trustworthy/dominant; 3: trustworthy/dominant; and 4: very trustworthy/dominant. Each image was presented for 1.5 s at the center of the screen.

10.1523/ENEURO.0398-21.2021.f1-1 Extended Data Figure 1-1 Spike sorting and recording quality assessment. ***A***, Histogram of the number of units identified on each active wire (only wires with at least one unit identified are counted). The average yield per wire with at least one unit was 2.54 ± 1.62 (mean ± SD). ***B***, Histogram of mean FRs. ***C***, Histogram of proportion of interspike intervals (ISIs), which are shorter than 3 ms. The large majority of clusters had less than 0.5% of such short ISIs. ***D***, Histogram of the signal-to-noise ratio (SNR) of the mean waveform peak of each unit. ***E***, Histogram of the SNR of the entire waveform of all units. ***F***, Pairwise distance between all possible pairs of units on all wires where more than one cluster was isolated. Distances are expressed in units of SD after normalizing the data such that the distribution of waveforms around their mean is equal to 1. ***G***, Isolation distance of all units for which this metric was defined (*n *=* *630, median = 14.56). ***H***, Neurons recorded in the trustworthiness judgment task versus dominance judgment task did not differ significantly in isolation distance (*t*_(534)_ = 0.76, *p* = 0.45). Download Figure 1-1, EPS file.

**Figure 2.**
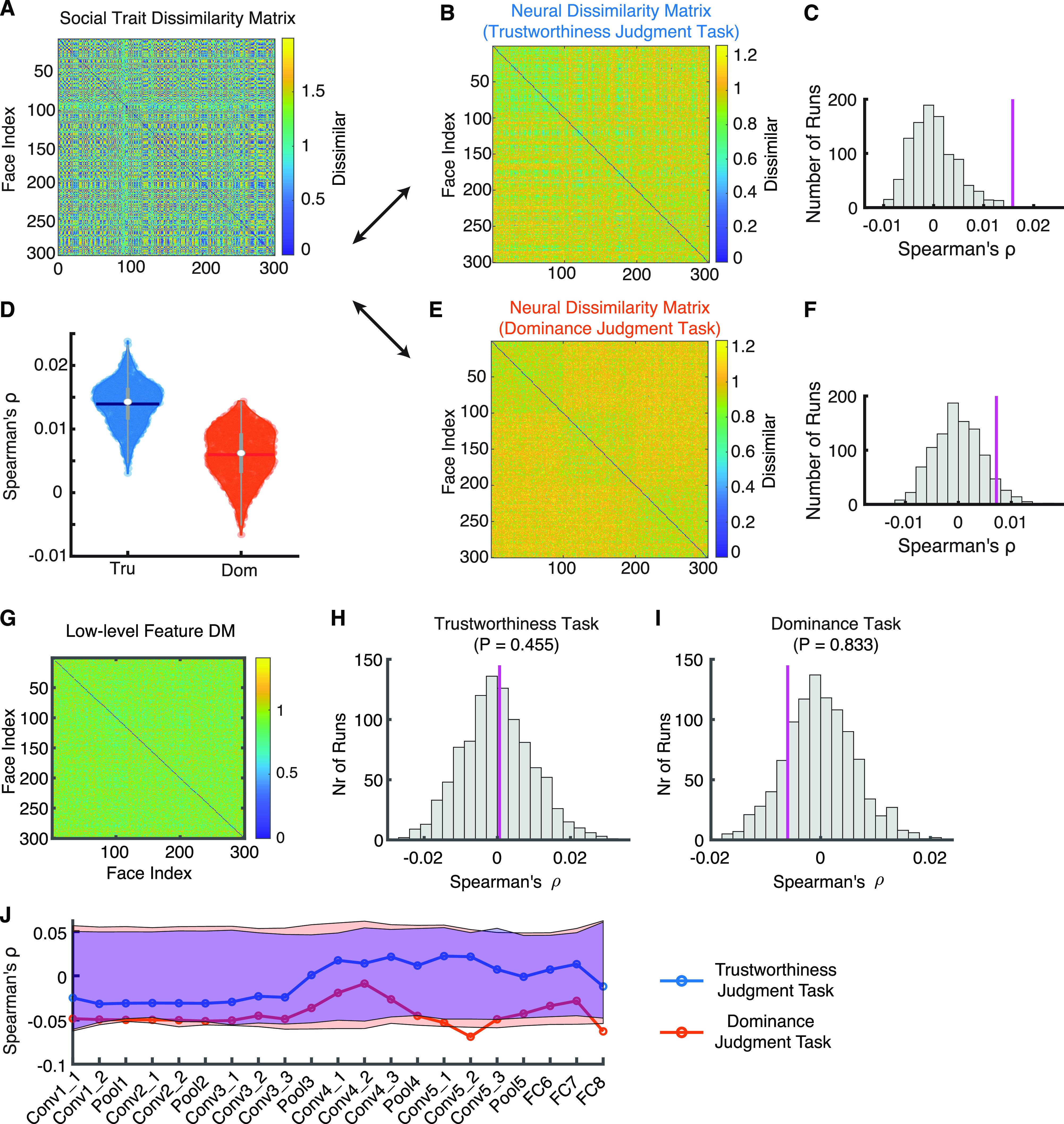
RSA for social trait and neural response. ***A***, The social trait DM. ***B***, ***E***, The neural response DM from (***B***) the trustworthiness judgment task and (***E***) the dominance judgment task. Color coding shows dissimilarity values. ***C***, ***F***, Observed versus permuted correlation coefficient between DMs. ***C***, Correlation between the social DM and the neural DM from the trustworthiness judgment task. ***F***, Correlation between the social DM and the neural DM from the dominance judgment task. The correspondence between DMs was assessed using permutation tests with 1000 runs. The magenta line indicates the observed correlation coefficient between DMs. The null distribution of correlation coefficients (shown in gray histogram) was calculated by permutation tests of shuffling the faces (1000 runs). ***D***, Bootstrap distribution of DM correspondence for each task instruction. Tru: trustworthiness judgment task (blue). Dom: dominance judgment task (red). Violin plots present the median value as the white circle and the interquartile range as the gray vertical bars. The neural response from the trustworthiness judgment task had a stronger correspondence with the social trait space compared with the neural response from the dominance judgment task. ***G–I***, RSA for neural response and low-level features. ***G***, The low-level feature DM. ***H***, ***I***, Observed versus permuted correlation coefficient between DMs. ***H***, Correlation between the low-level feature DM and the neural DM from the trustworthiness judgment task. ***I***, Correlation between the low-level feature DM and the neural DM from the dominance judgment task. The correspondence between DMs was assessed using permutation tests with 1000 runs. The magenta line indicates the observed correlation coefficient between DMs. The null distribution of correlation coefficients (shown in gray histogram) was calculated by permutation tests of shuffling the faces (1000 runs). ***J***, Correlation between the DNN feature DM and the neural DM. Null distribution was estimated using 1000 permutation runs. Red, trustworthiness judgment task; blue, dominance judgment task. Shaded area denotes the statistical significance threshold (95% interval of the null distribution).

10.1523/ENEURO.0398-21.2021.f1-2 Extended Data Figure 1-2 Control analyses for choices. ***A***, Distribution of choices across tasks. Blue, trustworthiness judgment task; red, dominance judgment task. Error bars denote 1 SEM across sessions. There was no significant difference between tasks for each choice (all *p*s > 0.1). ***B***, An example neuron showing a significant correlation with choices in the trustworthiness judgment task. ***C***, An example neuron showing a significant correlation with choices in the dominance judgment task. Error bars denote ±SEM across images. ***D***, Percentage of neurons showing a significant correlation with choices (trustworthiness judgment task: *n *=* *21, 7.24%, binomial *p* = 0.04; dominance judgment task: *n *=* *34, 10%, binomial *p* < 0.0001). The percentages did not differ significantly between tasks (χ^2^ test: *p* = 0.22). ***E***, Overlap of choice neurons between tasks with neurons recorded from both tasks. The overlap between choice neurons across tasks was not above chance (χ^2^ test; trustworthiness judgment task: *p* = 0.14; dominance judgment task: *p* = 0.60). ***F***, Correlation between neural response and patients’ social judgment ratings. ***G***, Correlation between neural response and social judgment ratings after excluding choice neurons. Bars show the mean correlation coefficient under each task instruction. Error bars denote ±SEM across neurons. Asterisks indicate a significant difference from 0 (two-tailed paired *t* test) or between conditions (two-tailed two-sample *t* test); *p < 0.05 and **p < 0.01. For each trait (trustworthiness or dominance), the left bars show the congruent conditions (i.e., the evaluated traits were the same as the task instruction) and the right bars show the incongruent conditions (i.e., the evaluated traits were different to the task instruction). Download Figure 1-2, EPS file.

### Electrophysiology

We recorded from implanted depth electrodes in the amygdala and hippocampus from patients with pharmacologically intractable epilepsy. Target locations in the amygdala and hippocampus were verified using postimplantation structural MRIs. At each site, we recorded from eight 40-μm microwires inserted into a clinical electrode as described previously (Rutishauser et al., 2006a, 2010). Efforts were always made to avoid passing the electrode through a sulcus, and its attendant sulcal blood vessels, and thus the location varied but was always well within the body of the targeted area. Microwires projected medially out at the end of the depth electrode and examination of the microwires after removal suggests a spread of ∼20–30°. The amygdala electrodes were likely sampling neurons in the mid-medial part of the amygdala and the most likely microwire location is the basomedial nucleus or possibly the deepest part of the basolateral nucleus. Bipolar wide-band recordings (0.1–9000 Hz), using one of the eight microwires as reference, were sampled at 32 kHz and stored continuously for off-line analysis with a Neuralynx system. The raw signal was filtered with a zero-phase lag 300- to 3000-Hz bandpass filter and spikes were sorted using a semiautomatic template matching algorithm as described previously (Rutishauser et al., 2006b). Units were carefully isolated and recording and spike sorting quality were assessed quantitatively (Extended Data Fig. 1-1).

### Eye tracking

Patients were recorded with a remote noninvasive infrared Eyelink 1000 system (SR Research). One of the eyes was tracked at 500 Hz. The eye tracker was calibrated with the built-in nine-point grid method at the beginning of each block. Fixation extraction was conducted using software supplied with the Eyelink eye tracking system. Saccade detection required a deflection of >0.1°, with a minimum velocity of 30°/s and a minimum acceleration of 8000°/s^2^, maintained for at least 4 ms. Fixations were defined as the complement of a saccade, i.e., periods without saccades. Analysis of the eye movement record was conducted off-line after completion of the experiments.

We excluded five sessions that had fewer than 10 fixations onto each facial region of interest (ROI) because of a substantial amount of missing eye tracking data, resulting in a total of 19 sessions for eye movement analysis.

To quantitatively compare the fixation densities within certain parts of the face, we defined three ROIs: eyes, mouth, and nose. Each ROI is a rectangle and the eye and mouth ROI have the same size. The fixation density was calculated for each participant during the entire 1 s stimulus period, and was normalized within each participant. Fixation locations were smoothed using a 2D Gaussian kernel (30 × 30 pixels) with a SD of three pixels.

### Data analysis: behavior

For behavioral data, we calculated rating consistency for each individual by correlating his/her ratings with the average ratings from the previous study (Oosterhof and Todorov, 2008), which served as the benchmark ratings. Since it has been reported that consensus ratings predict neural responses better than individual ratings (Engell et al., 2007), here we used the average ratings from (Oosterhof and Todorov, 2008) for further analysis.

### Data analysis: spikes

Consistent with our previous studies (Wang et al., 2014, 2017, 2018b; Cao et al., 2020b, 2021a, b), only single units with an average firing rate (FR) of at least 0.15 Hz throughout the entire task were considered. Trials were aligned to stimulus onset. We used the mean FR in a time window 250–1750 ms after stimulus onset as the response to each face. Fixations were aligned to fixation onset. We used the mean FR in a time window from 200 ms before fixation onset to 200 ms after fixation offset as the response to each fixation.

### Data analysis: representational similarity analysis (RSA)

Dissimilarity matrices (DMs; Kriegeskorte et al., 2008) are symmetrical matrices of dissimilarity between all pairs of faces. In a DM, larger values represent larger dissimilarity of pairs, such that the smallest value possible is the similarity of a condition to itself (dissimilarity of 0). We used the Pearson correlation to calculate DMs (ratings were *z*-scored and FRs were normalized to the mean baseline of each neuron), and we used the Spearman correlation to calculate the correspondence between the DMs (Spearman correlation was used because it does not assume a linear relationship; Stolier and Freeman, 2016). We further used permutation tests with 1000 runs to assess the significance of the correspondence between the social trait DM and the neural response DM. We compared the distribution of the correspondence between DMs (Spearman’s ρ) computed with shuffling (i.e., null distribution) with the one without shuffling (i.e., observed response) to derive statistical significance.

To compare between task instructions, we used a bootstrap with 1000 runs to estimate the distribution of DM correspondence for each task instruction. In each run, 70% of the data were randomly selected from each task instruction and we calculated the correspondence (Spearman’s ρ) between the social trait DM and the neural response DM for each task instruction. We then created a distribution of DM correspondence for each task instruction, and we compared the means of the distributions to derive statistical significance.

### Data analysis: axis-based feature coding

To identify neurons that encoded a linear combination of facial features, we employed a simple linear regression with all 100 features, a partial least squares (PLS) regression with five components (Yamins et al., 2014; Ponce et al., 2019), and a classic face model with all 100 features (Oosterhof and Todorov, 2008; Chang and Tsao, 2017). Because only the linear regression model’s performance could be assessed directly using *R*^2^, for the classic face model and PLS regression model, we used the Pearson correlation between the predicted and actual neural response in the test dataset to assess model predictability (the same procedure as the permutation test below). For the classic face model, we fitted a linear model for the mean FRs and calculated the vector of feature weights *w* as: *w *=* F* • *r,* where *r* is a column vector (*N *×* *1) of the FRs to the *N* faces, and *F* is the feature matrix (each row is a feature and each column is a face) that contains the feature values for each face. We further normalized *w* by ‖*w*‖: *w *=* w*/‖*w*‖. The resulting feature vector *w* thus showed the optimal direction that best captured the variation in neural response.

We used a permutation test with 1000 runs to determine whether a neuron encoded a significant linear model of low-level facial features (i.e., the neuron encoded a linear combination of features of the face space). In each run, we randomly shuffled face labels and used 70% of the faces as the training dataset. We used the training dataset to construct a model (i.e., deriving regression coefficients), predicted responses using this model for each face in the remaining 30% of faces (i.e., test dataset), and computed the Pearson correlation between the predicted and actual response in the test dataset. The distribution of correlation coefficients computed with shuffling (i.e., null distribution) was eventually compared with the one without shuffling (i.e., observed response). If the correlation coefficient of the observed response was >95% of the correlation coefficients from the null distribution, this face model was considered significant. This procedure has been shown to be very effective selecting units with significant face models (Chang and Tsao, 2017). Note that the correlation coefficient between the predicted and actual response could index the model’s predictability and be compared statistically.

### Data analysis: region-based feature coding

To select neurons that demonstrated region-based feature coding (i.e., having elevated response for faces in a certain region of the face feature space), we first estimated a continuous spike density map in the feature space by smoothing the discrete FR map using a 2D Gaussian kernel (kernel size = feature dimension range * 0.2, SD = 4). We then estimated statistical significance for each pixel by permutation testing: in each of the 1000 runs, we randomly shuffled the labels of faces. We calculated the *p* value for each pixel by comparing the observed spike density value to those from the null distribution derived from permutation. We lastly selected the region with significant pixels (permutation *p* < 0.01, cluster size >1.8% of the total number of pixels of the face space). We also applied a mask to exclude pixels from the edges and corners of the spike density map where there were no faces because these regions were susceptible to false positives given our procedure. If a neuron had a region with significant pixels, the neuron was defined as a “region-coding neuron” and demonstrated “region-based feature coding.”

### Data analysis: response index for single fixation

For each neuron we quantified whether its response differed between fixation on the eyes and fixation on the mouth using a single-fixation selectivity index (FSI; Eq. 1). The FSI facilitates group analysis and comparisons between different types of cells (i.e., eyes-preferring and mouth-preferring cells in this study), as motivated by previous studies (Wang et al., 2014, 2018b). The FSI quantifies the response during fixation *i* relative to the mean response to fixations on the mouth and baseline (the interval right before face onset). The mean response and baseline were calculated individually for each neuron.

(1) $$FSI_{i}=\frac{FR_{i}-mean(FR_{Mouth})}{mean(FR_{Baseline})}\cdot 100\%.$$

For each fixation *i*, *FSI_i_* is the baseline normalized mean FR during an interval from 200 ms before fixation onset to 200 ms after fixation offset (the same time interval as cell selection). Different time intervals were tested as well, to ensure that results were qualitatively the same and not biased by particular spike bins.

If a neuron distinguishes fixations on the eyes from fixations on the mouth, the average value of *FSI_i_* of all fixations will be significantly different from 0. Since eyes-preferring neurons have more spikes in fixations on the eyes and mouth-preferring neurons have more spikes in fixations on the mouth, on average *FSI_i_* is positive for eyes-preferring neurons and negative for mouth-preferring neurons. To get an aggregate measure of activity that pools across neurons, *FSI_i_* was multiplied by −1 if the neuron is classified as a mouth-preferring neuron (Eq. 2). This makes *FSI_i_* on average positive for both types of eye-mouth-selective neurons. Notice that the factor −1 depends only on the neuron type but not fixation type. Thus, negative *FSI_i_* values are still possible.

(2) $$FSI_{i}=-\frac{FR_{i}-mean(FR_{Mouth})}{mean(FR_{Baseline})}\cdot 100\%.$$

After calculating *FSI_i_* for every fixation, we subsequently averaged all *FSI_i_* of fixations that belong to the same category. By definition, the average value of *FSI_i_* for fixation on the mouth will be equal to zero because the definition of *FSI_i_* is relative to the response to fixation on the mouth (see Eq. 2). The mean baseline FR was calculated across all trials. The same *FR_Mouth_* was subtracted for both types of fixations.

The cumulative distribution function (CDF) was constructed by calculating for each possible value *x* of the FSI how many examples are smaller than *x*. That is, *F*_(x)_ = P(*X *≤* x*), where *X* is a vector of all FSI values. The CDF of fixations on the eyes and mouth were compared using two-tailed two-sample Kolmogorov–Smirnov (KS) tests.

### Code accessibility

The code described in this paper is publicly available on OSF (https://osf.io/2xrgd/).

## Results

### Task modulation of neural encoding of social traits

We employed a social judgment task where participants rated the level of perceived trustworthiness or dominance of each face (Fig. 1*A*). The same 300 faces were rated in separate tasks for trustworthiness and dominance (order randomized). There were 12 sessions of trustworthiness judgment task and 12 sessions of dominance judgment task (Table 1). Behaviorally, the ratings from our neurosurgical patients were consistent with the prior report (Oosterhof and Todorov, 2008): we found that both trustworthiness (two-tailed paired *t* test of correlation coefficient against 0: *t*_(11)_ = 5.84, *p* = 1.13 × 10^−4^; Fig. 1*B*) and dominance (*t*_(11)_ = 7.72, *p* = 9.19 × 10^−6^; Fig. 1*B*) ratings were significantly correlated with the consensus ratings. Therefore, we next used the consensus ratings to analyze the neural response.

We recorded in total 857 neurons from the amygdala and hippocampus and we restricted our analysis to a subset of 630 neurons that had an overall FR > 0.15 Hz (Table 1). Among these neurons, 328 neurons were from the amygdala and 302 neurons were from the hippocampus (Table 1). Furthermore, 290 neurons were recorded from the trustworthiness judgment task and 340 neurons were recorded from the dominance judgment task (Table 1). We used the mean FR in a time window from 250 to 1750 ms after stimulus onset as the response.

We first analyzed the correlation between the neural response and the social traits of trustworthiness/dominance, and compared the correlation strength between the congruent (i.e., the response to the trustworthiness trait in the trustworthiness judgment task and the response to the dominance trait in the dominance judgment task) and incongruent (i.e., the response to the trustworthiness trait in the dominance judgment task and the response to the dominance trait in the trustworthiness judgment task) conditions. We found that neurons at the population level encoded the level of trustworthiness and dominance in the congruent conditions (Fig. 1*G*; see Fig. 1*C*,*D* for single-neuron examples), and notably, the response differed between congruent and incongruent conditions (Fig. 1*G*), suggesting that task instructions modulated the neural encoding of social traits. This was primarily the case for the trustworthiness trait [congruent: Pearson’s *r *=* *0.0076 ± 0.066 (mean ± SD across neurons); incongruent: *r* = −0.0075 ± 0.062; two-tailed paired *t* test: *t*_(628)_ = 2.96, *p* = 0.003]; the dominance trait only showed a marginally significant difference between conditions (congruent: *r* = −0.0073 ± 0.060; incongruent: *r *=* *0.0015 ± 0.060; *t*_(628)_ = 1.82, *p* = 0.07). Notably, a subset of 143 neurons were recorded with both task instructions, and we confirmed that the neuronal population coding of the trustworthiness trait (congruent: Pearson’s *r *=* *0.017 ± 0.062; incongruent: *r* = −0.0087 ± 0.062; two-tailed paired *t* test: *t*_(142)_ = 3.65, *p* = 3.68 × 10^−4^; Fig. 1*H*) but not the dominance trait (congruent: *r* = −0.011 ± 0.054; incongruent: *r *=* *0.0061 ± 0.061; *t*_(142)_ = 0.71, *p* = 0.48; Fig. 1*H*) was modulated by task instructions. A scatterplot of correlation coefficient between congruent versus incongruent conditions further showed a dissociation between task instructions (Pearson’s correlation of correlation coefficient between congruent vs incongruent tasks: trustworthiness trait: *r*_(143)_ = 0.013, *p* = 0.88; dominance trait: *r*_(143)_ = 0.098, *p* = 0.25; Fig. 1*E*,*F*).

We conducted three control analyses. First, we found similar results with face-responsive neurons only (i.e., neurons that significantly changed response compared with the baseline). Second, we found consistent results using each patient’s own trustworthiness/dominance ratings (Extended Data Fig. 1-2*F*). Notably, we confirmed that patients’ rating choices did not differ between tasks (Extended Data Fig. 1-2*A*), suggesting that our results could not be simply attributed to a choice bias between tasks; and we identified a subset of neurons that encoded rating choices (Extended Data Fig. 1-2*B–D*), which were further modulated by task instructions (Extended Data Fig. 1-2*E*). Importantly, we found a similar pattern of results as in Figure 1*G* when we excluded these choice neurons (Extended Data Fig. 1-2*G*). Third, we found that neurons recorded in the trustworthiness judgment task and dominance judgment task did not differ significantly in spike sorting qualities (Extended Data Fig. 1-1*H*).

Do amygdala and hippocampal neurons respond differently to social trait judgments? And are amygdala and hippocampal neurons modulated differently by task instructions? We next investigated the population encoding of social traits separately for amygdala and hippocampal neurons. We found that amygdala neurons only encoded the trustworthiness trait (Fig. 1*I*), whereas hippocampal neurons only encoded the dominance trait (Fig. 1*J*) in the congruent tasks. Notably, task instructions modulated the encoding of the trustworthiness and dominance traits in the amygdala (*t*_(326)_ = 4.16, *p* < 0.0001; Fig. 1*I*) and hippocampus (*t*_(300)_ = 2.34, *p* = 0.02; Fig. 1*J*), respectively. Together, our results have revealed different roles of the amygdala and hippocampus in coding social traits.

Lastly, we investigated whether neurons in the amygdala and hippocampus represented the social trait space as a whole (i.e., neurons collectively encoded multiple social traits and their similarities) and whether task instructions modulated the encoding of the social trait space. We employed a RSA (Kriegeskorte et al., 2008) and constructed the social trait DM (Fig. 2*A*) using consensus ratings of nine social traits (Oosterhof and Todorov, 2008). We constructed neural response DMs separately for the trustworthiness (Fig. 2*B*) and dominance (Fig. 2*E*) judgment tasks. The correspondence between the neural response DM and the social trait DM was then calculated for each task and the significance of the correspondence was estimated using a permutation test by shuffling the face labels (see Materials and Methods). We found that the neural response from the trustworthiness judgment task (ρ = 0.016, permutation *p* = 0.003; Fig. 2*C*) but not the dominance judgment task (ρ = 0.007, permutation *p* = 0.06; Fig. 2*F*) had a significant correspondence with the social trait space. Indeed, the neural response from the trustworthiness judgment task had a stronger correspondence with the social trait representations compared with the dominance task (Fig. 2*D*; permutation test comparing the mean of the trustworthiness judgment task to the distribution of the dominance judgment task: *p* = 0.002; permutation test comparing the mean of the dominance judgment task to the distribution of the trustworthiness judgment task: *p* = 0.015), suggesting that task instructions impacted on the holistic encoding of the social trait space structure.

Could the neural response DM be explained by low-level features as well? To answer this question, we constructed a linear regression model that used both the social trait DM and the low-level feature DM to explain the neural response DM (the low-level feature DM was calculated using the 50 shape features and 50 texture features; see Materials and Methods; Fig. 2*G*). We first confirmed that the regression coefficient for the social trait DM was significant for the trustworthiness judgment task (β = 0.0024, *p* = 0.0016) but not the dominance judgment task (β = 0.0012, *p* = 0.067). Interestingly, we found that the regression coefficient for the low-level feature DM was not significant for the trustworthiness judgment task (β = −0.002, *p* = 0.61) but significant for the dominance judgment task (β = −0.008, *p* = 0.038), although a direction comparison showed that that the neural response from the trustworthiness judgment task (ρ = 0.0006, permutation *p* = 0.46; Fig. 2*H*) and the dominance judgment task (ρ = −0.006, permutation *p* = 0.83; Fig. 2*I*) did not have a significant correspondence with the low-level feature space. Therefore, low-level features were limited in explaining the neural response DM. Furthermore, we used a pretrained deep neural network (DNN) trained for face identification (Parkhi et al., 2015; see also Cao et al., 2020b) to extract features from faces (note that such DNN has been shown to contain information about social traits; Parde et al., 2019; Lin et al., 2021). We found that for both trustworthiness judgment task and dominance judgment task, the neuronal population did not show a significant correspondence with DNN layers (Fig. 2*J*), confirming that the neural response DM was not likely explained by low-level features.

Together, our results suggest that tasks and evaluative contexts modulate the neural encoding of social traits.

### Task modulation of neural encoding of low-level facial features

We next investigated whether amygdala and hippocampal neurons encoded low-level facial features (i.e., physical changes in the face such as face shape or skin tone) and whether encoding of low-level facial features was modulated by task instructions.

**Figure 3.**
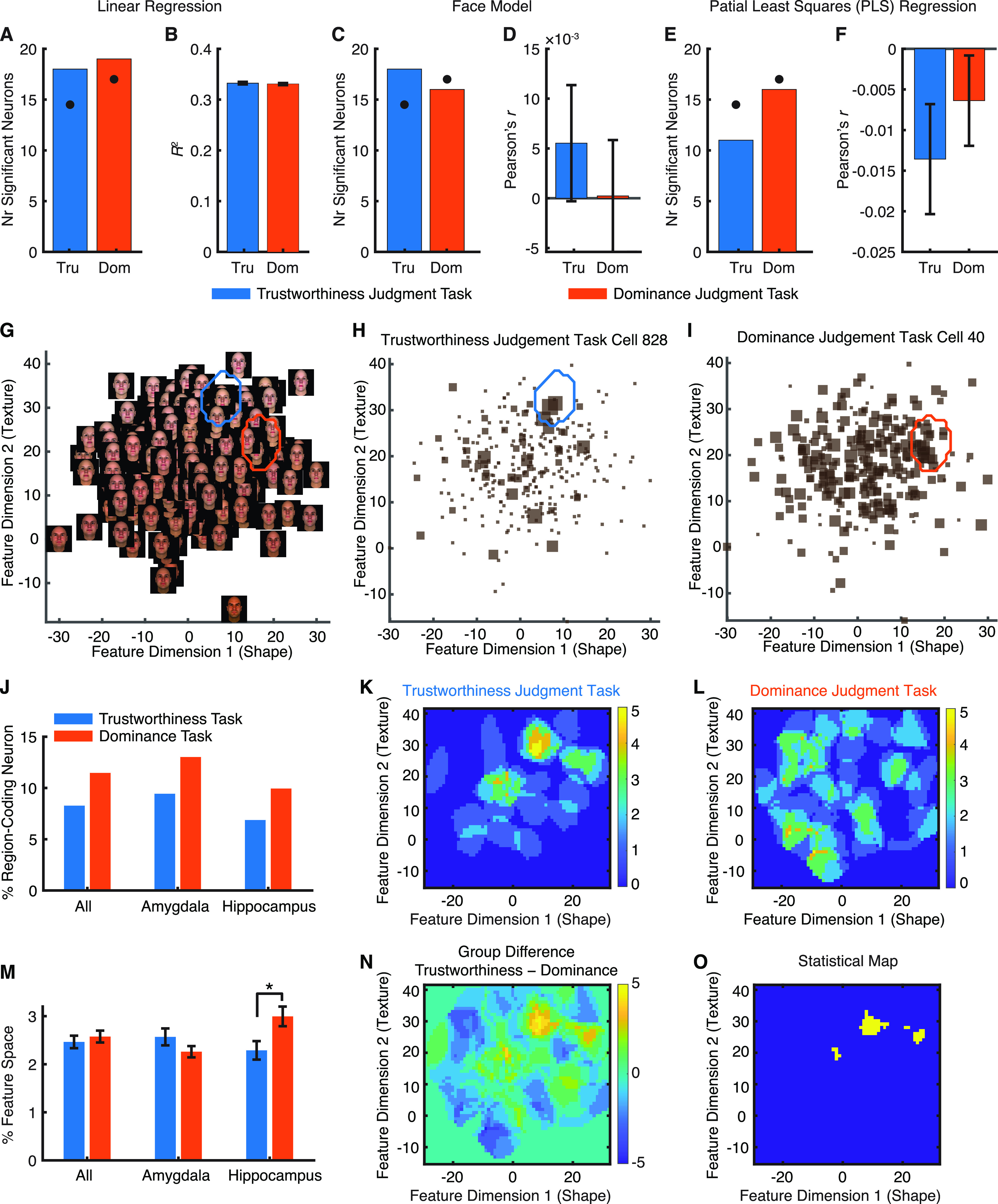
Task modulation of neural encoding of low-level facial features. ***A–F***, Axis-based feature coding. ***G–M***, Region-based feature coding. ***A***, ***B***, A simple linear regression model. ***C***, ***D***, A classic face model. ***E***, ***F***, A PLS regression model. ***A***, ***C***, ***E***, The number of neurons showing a significant face model. Black dots show the chance number of significant neurons (5% of all neurons). ***B***, ***D***, ***F***, Model assessment. For the classic face model and PLS regression model, model predictability was assessed using the Pearson correlation between the predicted and actual neural response in the test dataset. Error bars denote ±SEM across neurons. ***G***, Feature space constructed by the first shape and tone/texture features that were used to generate the stimuli. Note that face shape varied along feature dimension 1 and skin color varied along feature dimension 2. ***H***, ***I***, Example neurons demonstrating region-based feature coding. The size of the squares indicates the FR. The blue/red outlines delineate the tuning regions of the neurons in the feature space. ***H***, An example neuron from the trustworthiness judgment task. ***I***, An example neuron from the dominance judgment task. ***J***, Percentage of neurons (all neurons, amygdala neurons, and hippocampal neurons) demonstrating region-based feature coding. ***K***, ***L***, The aggregated tuning regions of the neuronal population. Color bars show the counts of overlap between individual tuning regions. ***K***, Trustworthiness judgment task. ***L***, Dominance judgment task. ***M***, The percentage of the feature space covered by the tuning regions did not differ between the trustworthiness judgment task and the dominance judgment task for all neurons and amygdala neurons. Error bars denote ±SEM across neurons. Asterisk indicates a significant difference between conditions (two-tailed two-sample t test); *p < 0.05. ***N***, Group difference in the aggregated tuning regions of the neuronal population (trustworthiness − dominance). Yellow, higher counts in the trustworthiness judgment task; blue, higher counts in the dominance judgment task; green, no difference between tasks. ***O***, Statistical map shows areas that had a significant difference in the proportion of neurons (i.e., the number of neurons that encoded a particular pixel divided by the total number of region-coding neurons) between tasks (yellow; χ^2^ test at each pixel, *p* < 0.05 uncorrected for multiple comparisons). Extended Data Figure 3-1 shows axis-based coding of low-level features analyzed separately for shape features and texture features. Extended Data Figure 3-2 shows region-based coding of low-level features analyzed separately for shape features and texture features.

First, to investigate whether amygdala and hippocampal neurons encode a linear combination of low-level facial features as shown in the primate inferotemporal cortex (IT; Chang and Tsao, 2017; Ponce et al., 2019), we employed three linear models, including a simple linear regression model [Fig. 3*A*,*B*; number of significant neurons (see Materials and Methods): *n *=* *18, 6.21%, binomial test on the number of significant neurons against chance-level selection (5%): *p* = 0.14 for the trustworthiness judgment task and *n *=* *19, 5.59%, binomial *p* = 0.26 for the dominance judgment task], a classic face model (Oosterhof and Todorov, 2008; Chang and Tsao, 2017; *n *=* *18, 6.21%, binomial *p* = 0.14 for the trustworthiness judgment task and *n *=* *16, 4.71%, binomial *p* = 0.53 for the dominance judgment task; Fig. 3*C*,*D*), and a PLS regression model (Yamins et al., 2014; Ponce et al., 2019; *n *=* *11, 3.79%, binomial *p* = 0.79 for the trustworthiness judgment task and *n *=* *16, 4.71%, binomial *p* = 0.53 for the dominance judgment task; Fig. 3*E*,*F*). For each model, we did not succeed at selecting a larger than expected by chance number of neurons that encoded a linear combination of low-level facial features (Fig. 3*A*,*C*,*E*). Furthermore, the neural response could not be predicted by a weighted sum of low-level facial features (Fig. 3*B*,*D*,*F*). We further replicated our results with shape features only (Extended Data Fig. 3-1) and texture features only (Extended Data Fig. 3-1), and we did not succeed at selecting a larger than expected by chance number of neurons for individual features (including the major shape features, the major texture features, and a combination of these; all binomial *p*s > 0.05). Therefore, amygdala and hippocampal neurons did not parametrically correlate with low-level facial features along specific axes in the face space (i.e., demonstrating axis-based feature coding). This is consistent with our previous neuroimaging finding that the amygdala does not encode a linear combination of low-level features (Cao et al., 2020a).

10.1523/ENEURO.0398-21.2021.f3-1 Extended Data Figure 3-1 Axis-based coding of low-level features analyzed separately for shape features (***A*–*C***, ***G*–*I*)** and texture features **(*D–F***, ***J–L*)**. Legend conventions as in Figure 3*A–F*. Download Figure 3-1, EPS file.

10.1523/ENEURO.0398-21.2021.f3-2 Extended Data Figure 3-2 Region-based coding of low-level features analyzed separately for shape features (***A–D***) and texture features (***E–H***). Legend conventions as in Figure 3*G–O*. Download Figure 3-2, EPS file.

Second, our previous study has shown that amygdala and hippocampal neurons encode a region in the feature space and thus demonstrate region-based feature coding (Cao et al., 2020b). Here, we further investigated whether region-based feature coding was modulated by task instructions. Using the first shape and texture features to construct a two-dimensional face space (Fig. 3*G*), we found populations of neurons demonstrating region-based feature coding (Fig. 3*H*,*I* for examples) in both trustworthiness judgment task (*n *=* *24, 8.28%, binomial *p* = 0.006; Fig. 3*H*,*J*) and dominance judgment task (*n *=* *35, 10.3%, binomial *p* < 0.001; Fig. 3*I*,*J*). Importantly, the percentage of neurons demonstrating region-based feature coding did not differ between task instructions (χ^2^ test: *p* > 0.05 for each group; Fig. 3*J*) and the coded area by the neuronal population did not differ between task instructions (two-tailed two-sample *t* test: *t*_(61)_ = 0.15, *p* = 0.88; Fig. 3*M*), although the coded regions by the neuronal population differed slightly between task instructions (χ^2^ test at each pixel: *p* < 0.05 uncorrected for multiple comparisons; Fig. 3*K*,*L*,*N*,*O*), which was likely because different neurons were recorded under different task instructions and different neurons might have different tuning regions. Furthermore, we found similar results for the percentage of region-coding neurons within amygdala neurons only (χ^2^ test: *p* = 0.31; Fig. 3*J*) and within hippocampal neurons only (χ^2^ test: *p* = 0.35; Fig. 3*J*; see also Fig. 3*M* for the percentage of encoded feature space). In addition, we replicated our results within the shape space (Extended Data Fig. 3-2) and within the texture space (Extended Data Fig. 3-2).

Together, although axis-based coding and region-based coding models were not directly comparable because of different neuron selection procedures and criteria (note that this study was not to compare these models but to compare task instructions under each model), we only found region-based coding in the human amygdala and hippocampus, likely because the selection of neurons for axis-based coding was more conservative (cross-validated using out-of-sample data, in contrast to the selection of neurons for region-based coding that was based entirely on within-sample data). We further showed that region-based coding of low-level facial features was not modulated by task instructions or evaluative contexts.

### Task modulation of eye movement and face part selectivity

**Figure 4.**
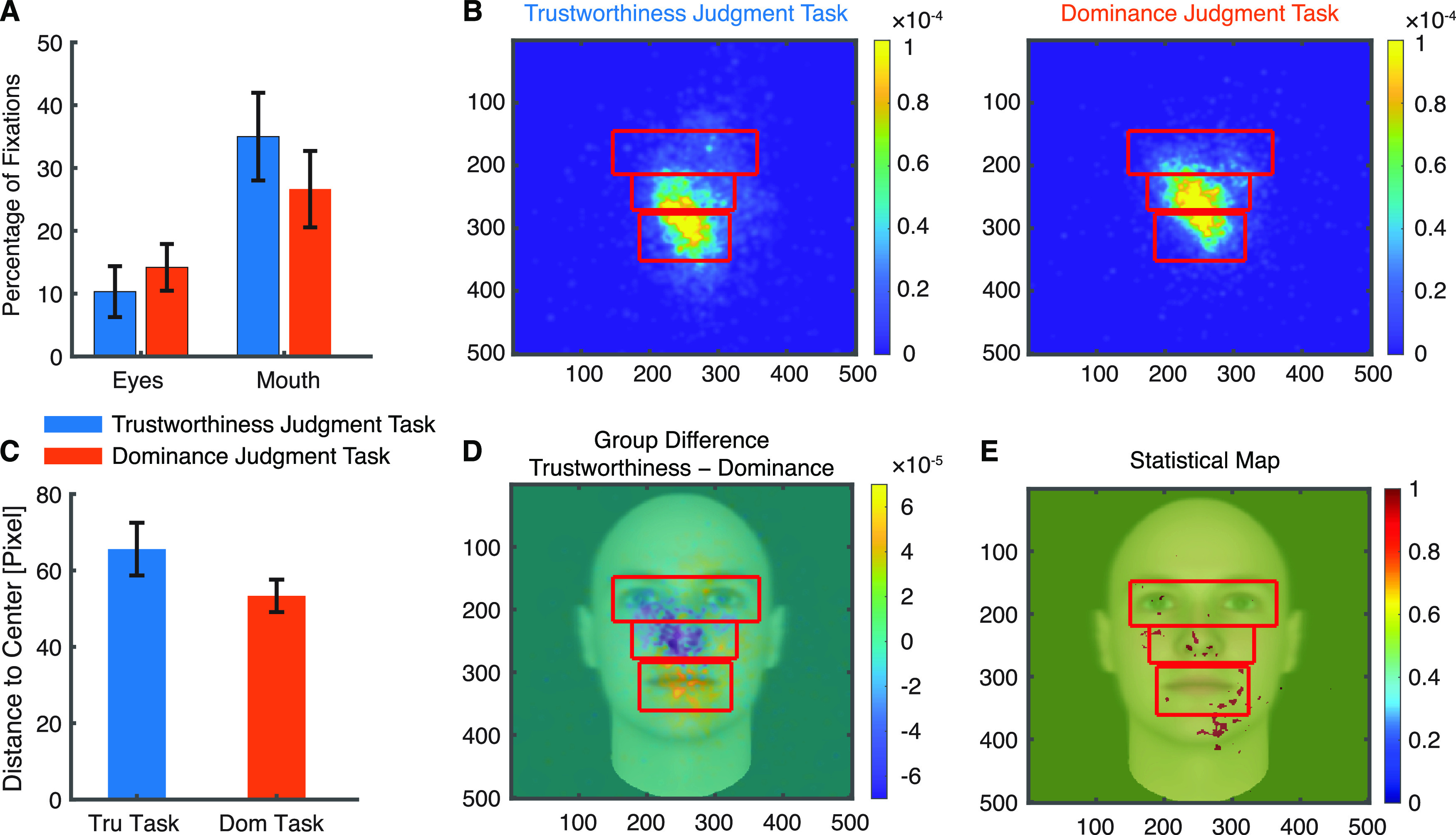
Task modulation of eye movement onto faces. ***A***, Percentage of fixations for each ROI. Error bars denote ±SEM across sessions. ***B***, Fixation density maps to quantify eye movements for different tasks. Each map shows the probability of fixating a given location within the entire stimulus period. The scale bar (color bar) is in arbitrary units. The ROIs (eye, mouth, nose) used for analysis are shown in red (not shown to patients). ***C***, Mean distance to the stimulus center (in pixels). Tru: trustworthiness. Dom: dominance. ***D***, Group difference density map (trustworthiness − dominance) shows areas that patients fixated more in the trustworthiness judgment task (yellow), and vice versa (blue), with green meaning there was no difference between tasks. ***E***, Statistical map shows areas that had a significant difference in density maps between tasks (red; two-tailed two-sample *t* test between individual density maps at each pixel, *p* < 0.05 uncorrected).

It has been shown that people use different facial information in different tasks (Schyns et al., 2002). We next explored whether different task instructions could modulate eye movement on faces. We found that neurosurgical patients had a similar percentage of fixations on the eyes and mouth in different tasks (two-tailed paired *t* test, eyes: *t*_(17)_ = 0.74, *p* = 0.47, mouth: *t*_(17)_ = 0.94, *p* = 0.36; Fig. 4*A*). Consistently, we found that the overall fixation density distributed similarly across tasks (Fig. 4*B*) and the mean pixel-wise distance to the stimulus center was comparable between tasks (*t*_(17)_ = 1.55, *p* = 0.14; Fig. 4*C*). Furthermore, we did not find a significant difference when we compared the density maps pixel by pixel (Fig. 4*D*,*E*). Therefore, our results suggest that task instructions did not modulate the percentage of fixations onto salient face parts (e.g., mouth) or the overall distribution of fixations.

**Figure 5.**
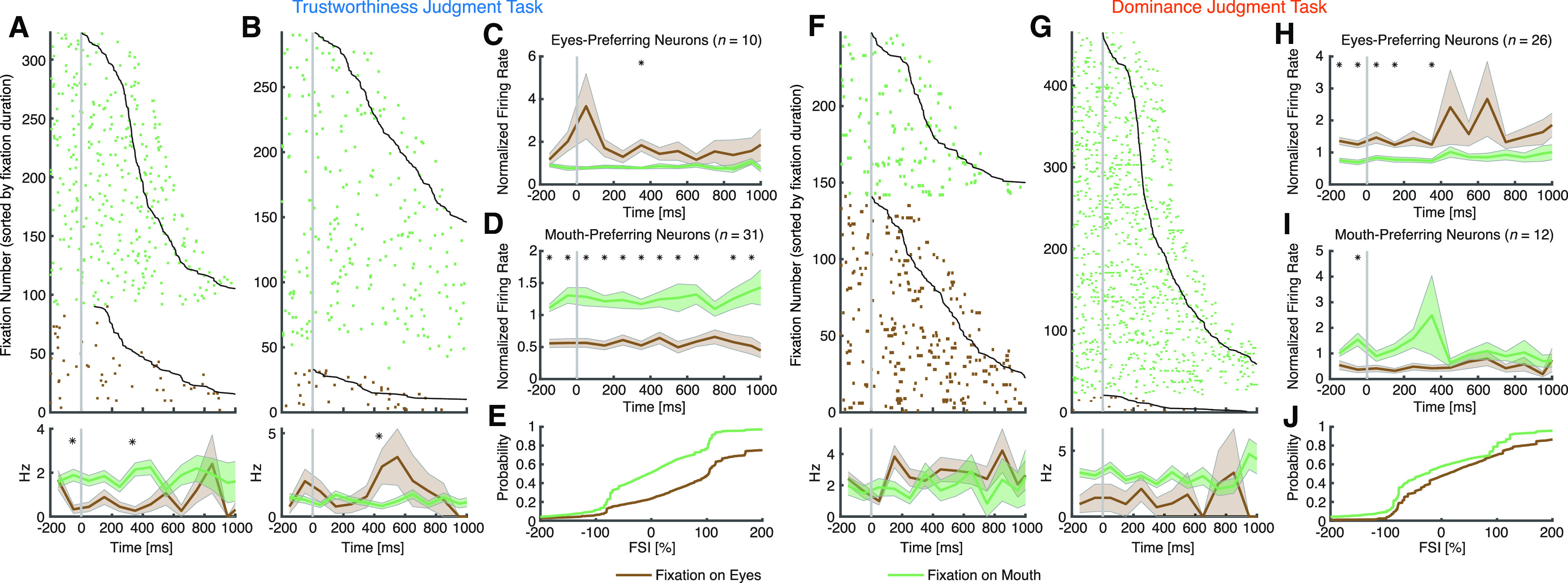
Task modulation of face part selectivity. ***A–E***, Trustworthiness judgment task. ***F–J***, Dominance judgment task. ***A***, ***F***, Neurons that had a significantly greater FR when fixating on the eyes compared with the mouth (selection by two-tailed *t* test in a time window of −200 ms before fixation onset to 200 ms after fixation offset). ***B***, ***G***, Neurons that had a significantly greater FR when fixating on the mouth compared with the eyes. Fixations are sorted by fixation duration (black line shows start of the next saccade). Fixation onset is *t* = 0. Asterisks indicate a significant difference between fixations on the eyes and mouth in that bin (*p* < 0.05, two-tailed *t* test, after Bonferroni correction; bin size = 100 ms). ***C*–*E***, ***H*–*J***, Population summary of all eye-mouth-selective. ***C***, ***H***, Average normalized FR of eyes-preferring neurons. ***D***, ***I***, Average normalized FR of mouth-preferring neurons. Shaded area denotes ±SEM across neurons. Asterisks indicate a significant difference between the fixation categories in that bin (*p* < 0.05, two-tailed *t* test, after Bonferroni correction). ***E***, ***J***, Single-fixation analysis using the FSI (Materials and Methods). Shown is the cumulative distribution of the single-fixation response of fixation-aligned eyes-preferring and mouth-preferring neurons for fixations on the eyes and mouth.

We next explored whether different task instructions modulated neural response to salient face parts such as the eyes and mouth. We found neurons that differentiated fixations onto the eyes or mouth in both tasks: we identified 41 neurons from the trustworthiness judgment task (15.8%; binomial *p* = 2.18 × 10^−11^; see Fig. 5*A*,*B* for examples and Fig. 5*C*,*D* for group results; note that two sessions with 31 neurons were excluded; see Materials and Methods) and 38 neurons from the dominance judgment task (13.6%; binomial *p* = 8.60 × 10^−9^; see Fig. 5*F*,*G* for examples and Fig. 5*H*,*I* for group results; note that three sessions with 61 neurons were excluded) differentiated fixations on the eyes versus the mouth. Notably, the percentage of significant neurons did not differ significantly between task instructions (χ^2^ test: *p* = 0.47). To investigate the relationship between the response of these eye-mouth-selective neurons and their behavior, we quantified the response of eye-mouth-selective neurons during individual fixations using a FSI (see Eqs. 1, 2; Materials and Methods). As expected, the FSI for eye-mouth-selective neurons was significantly larger during fixations on the eyes compared with fixations on the mouth (two-tailed two-sample KS test; trustworthiness: KS = 0.34, *p* = 1.30 × 10^−246^; dominance: KS = 0.17, *p* = 5.00 × 10^−70^; Fig. 5*E*,*J*). Notably, the FSI did not differ between tasks (two-tailed two-sample *t* test: *t*_(77)_ = 1.67, *p* = 0.10).

Similarly, we found that 26 neurons from the trustworthiness judgment task (9.92%; binomial *p* = 3.44 × 10^−4^) and 25 neurons from the dominance judgment task (8.96%; binomial *p* = 0.0019) encoded saccades to the eyes, and the percentage did not differ significantly between task instructions (χ^2^ test: *p* = 0.68). Furthermore, we found that 18 neurons from the trustworthiness judgment task (6.87%; binomial *p* = 0.069) and 22 neurons from the dominance judgment task (7.89%; binomial *p* = 0.014) encoded saccades to the mouth, and the percentage did not differ significantly between task instructions (χ^2^ test: *p* = 0.68).

Together, we found that amygdala and hippocampal neurons encoded salient face parts such as the eyes and mouth in both tasks and their response to face parts was similar between tasks.

### Population summary

**Figure 6.**
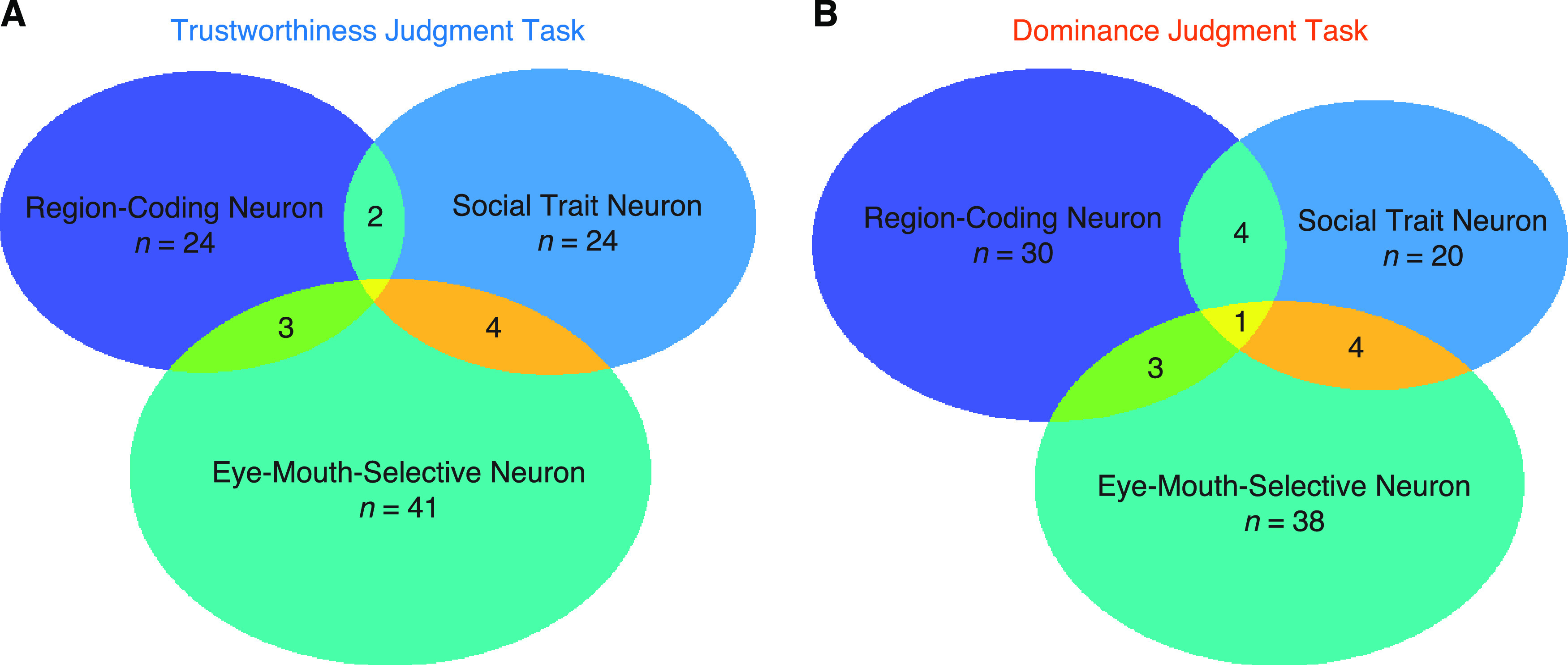
Summary of each type of neuron in the amygdala and hippocampus. ***A***, Trustworthiness judgment task. ***B***, Dominance judgment task.

Lastly, we summarized three subpopulations of neurons in the amygdala and hippocampus (Fig. 6). We found that neurons that encoded social traits (i.e., trustworthiness and dominance in the congruent task) and neurons that demonstrated region-based feature coding were largely distinct, because the proportion of neurons that qualified as both was not greater than expected from independence of these two attributes (i.e., neurons that encoded social traits had a similar percentage of neurons that demonstrated region-based feature coding as the entire population), and this was the case for both trustworthiness judgment task (χ^2^ test: *p* = 0.99) and dominance judgment task (*p* = 0.83). We also found that neurons that encoded social traits and eye-mouth-selective neurons were largely distinct, for both trustworthiness judgment task (*p* = 0.23) and dominance judgment task (*p* = 0.54). Furthermore, we found that neurons that demonstrated region-based feature coding and eye-mouth-selective neurons were largely distinct, for both trustworthiness judgment task (*p* = 0.44) and dominance judgment task (*p* = 0.90). In addition, by randomly splitting the trials into three disjoint sets for selection of neurons for each attribute, we still found that the overlap between subpopulations of neurons was not greater than expected from independence (all *p*s > 0.05).

Although we expected an above-chance overlap of selected/significant neurons when we randomly split the trials into two disjoint sets for selection of neurons, we only found so for social trait neurons. This was likely because (1) it was not a sensitive analysis to use the overlapping numbers of selected neurons; (2) the thresholding for defining significant neurons was arbitrary (i.e., neurons may be at the borderline of significance so they may appear as significant in one split but nonsignificant in the other split, resulting in unstable classifications); and (3) for region-coding neurons, the distribution of faces in the face space was different given different split of trials (note that we used a spike density map to select region-coding neurons, and the spike density map may vary as a function of the configuration of the face space). To further demonstrate consistency of trials, we employed a more sensitive parametric approach: we correlated the correlation coefficient between splits of trials for neurons encoding social traits, and we correlated the FSI between splits of trials for neurons encoding eye movement. We found that both neurons encoding social traits (trustworthiness judgment task: *r *=* *0.76, *p* = 1.83 × 10^−5^; dominance judgment task: *r *=* *0.78, *p* = 5.38 × 10^−5^) and neurons encoding eye movement (trustworthiness judgment task: *r *=* *0.71, *p* = 2.14 × 10^−7^; dominance judgment task: *r *=* *0.74, *p* = 7.03 × 10^−8^) showed a significant correlation between splits of trials, suggesting consistency of trials (note that a metric to parametrically summarize the strength of region-based coding was not available given its two-dimensional nature). Notably, we found no significant correlation between the correlation coefficient of social traits and the FSI (trustworthiness judgment task: *r* = −0.076, *p* = 0.22; dominance judgment task: *r *=* *0.032, *p* = 0.60), confirming that these two attributes were encoded separately.

Together, the three subpopulations of neurons identified in this study were largely distinct, and they were subject to task modulation differentially.

## Discussion

In this study, we comprehensively analyzed different aspects of face processing, and in particular, for the first time analyzed context dependency of face perception at the single-neuron level in humans. Specifically, (1) we found that task instructions modulated encoding of social traits, consistent with our previous study showing that the amygdala has a flexible encoding of social traits (Cao et al., 2020a). (2) We found that neurons in the amygdala and hippocampus demonstrated region-based but not axis-based feature coding, consistent with our previous findings using natural real photos (Cao et al., 2020b). This is also consistent with our prior neuroimaging report that the amygdala does not demonstrate axis-based coding (Cao et al., 2020a). In particular, we showed that region-based feature coding was not modulated by task instructions or evaluative contexts. (3) We found that neurons in the amygdala and hippocampus encoded salient face parts, consistent with our prior study using natural real photos (Cao et al., 2021b). We further showed that task instructions did not modulate neuronal response to eye movements to face parts. Together, our results showed that some processes of face perception were modulated by task instructions but not others.

Because of the length of the experiment, most of the trustworthiness judgment tasks and dominance judgment tasks were recorded in different sessions. Therefore, most of the neurons under different tasks were not the same, and our conclusions were largely based on the neuron population statistics. However, we confirmed our results with a subset of neurons recorded for both tasks. Furthermore, although we only used computer-generated model faces in this study, encoding of social traits (Cao et al., 2021a), low-level facial features (Cao et al., 2020b), and eye movement (Cao et al., 2021b) can all be replicated using natural photographs of real human faces.

In each task, we found largely distinct subpopulations of neurons for each attribute (Fig. 6), consistent with our prior findings that only a small and distinct proportion (∼20%) of human amygdala and hippocampal neurons are involved in coding a certain task aspect, such as attention (Wang et al., 2018b, 2019), task sequence (Wang et al., 2019), visual category selectivity (Wang et al., 2018b), eye movement (Cao et al., 2021b), social judgment (Cao et al., 2021a), as well as face identity (Cao et al., 2020b), although other studies have suggested nested/mixed selectivity in the monkey amygdala (Gothard et al., 2007; Mosher et al., 2014). Mixed selectivity in the amygdala may be critical for flexible and context-dependent social behavior (Gothard, 2020); and the lack of mixed selectivity in our present and previous studies (Wang et al., 2018b, 2019) was likely because the task attributes under investigation were not closely related. A future study is therefore needed to systematically investigate multidimensional processing in the human amygdala and hippocampus.

Although our previous neuroimaging study has shown that there is a flexible encoding of social traits or low-level facial features in various face encoding areas including the amygdala (Cao et al., 2020a), it does not have the spatial and temporal resolution to tease apart different neural processes underlying face perception or separate the response from different neurons. Here, we not only showed that different neural processes were modulated by task instructions differently, but also demonstrated that different subpopulations of neurons encoded different processes of face perception. In line with this idea, our prior report showed that during approach versus avoidance decisions (rather than trustworthiness or dominance judgment as in the present study) the right amygdala’s response to trustworthiness is not modulated by stimulus range or social context (Wang et al., 2018a), although the decisions are based on facial trustworthiness.

Our present results suggest invariant encoding of low-level features but flexible encoding of social traits under different contexts in the human amygdala and hippocampus. On the one hand, the invariant coding of low-level facial features supports the functional role of the amygdala and hippocampus in invariant visual representation of face identities (Quian Quiroga et al., 2005; Quian Quiroga, 2012). On the other hand, the flexible coding of social traits is consistent with the idea that the hippocampus plays a critical role in flexible cognition and social behavior (Rubin et al., 2014). It has been shown that neurons in the amygdala and hippocampus flexibly encode task demands and are engaged in a flexible recruitment of memory-based choice representations (Minxha et al., 2020). Furthermore, the amygdala processes stimulus relevance and evaluative goals and it can be dynamically modulated by motivations of the perceiver (Cunningham et al., 2008; Cunningham and Brosch, 2012).

Top-down modulations in the amygdala and hippocampus may originate from the frontal cortex (Corbetta and Shulman, 2002; Zanto et al., 2011), which has extensive anatomic and functional connections with the amygdala (Amaral and Price, 1984; Gangopadhyay et al., 2021). The frontal cortex reads out context or motivation in perceptual decision-making (Gold and Shadlen, 2007). For example, the prefrontal cortex shows context-dependent neural computation when monkeys flexibly select and integrate noisy sensory inputs toward a choice (Mante et al., 2013), and the inferior frontal gyrus shows flexible neural coding during categorical decision-making by shaping its selectivity to reflect the behaviorally relevant features (Li et al., 2007). Besides, social judgments modulate the response to faces in the inferior frontal gyrus and dorsal medial prefrontal cortex (Bzdok et al., 2012). Many studies have implicated the frontal cortex in modulating face perception by integrating perceptual representations with top-down expectations activated by task instructions, task demands, and evaluative contexts (Ratner et al., 2013; Collins et al., 2016; Stolier and Freeman, 2016). In line with this idea, we have shown that the neural signature from the medial prefrontal cortex indexing facial ambiguity is modulated by context (Sun et al., 2017b) and task instructions (Sun et al., 2017a), which is in turn related to amygdala activity (Wang et al., 2017). We have also shown that the goal-directed attentional signal to faces in the amygdala and hippocampus (Wang et al., 2018b) may also originate from the medial frontal cortex (Wang et al., 2019).

## Synthesis

Reviewing Editor: Ifat Levy, Yale University School of Medicine Decisions are customarily a result of the Reviewing Editor and the peer reviewers coming together and discussing their recommendations until a consensus is reached. When revisions are invited, a fact-based synthesis statement explaining their decision and outlining what is needed to prepare a revision will be listed below. The following reviewer(s) agreed to reveal their identity: NONE. Note: If this manuscript was transferred from JNeurosci and a decision was made to accept the manuscript without peer review, a brief statement to this effect will instead be what is listed below.

Thanks for all your edits and hard work in addressing the concerns.

Please incorporate your answer to point 6 (i.e. selection of cells and regarding the validation of the chi-squared test of proportions) to the manuscript. This is an important caveat to the analysis and you need to make clear how you ensured that the results you present are indeed trustworthy.
